# High-Throughput MicroRNA and mRNA Sequencing Reveals That MicroRNAs May Be Involved in Melatonin-Mediated Cold Tolerance in *Citrullus lanatus* L.

**DOI:** 10.3389/fpls.2016.01231

**Published:** 2016-08-15

**Authors:** Hao Li, Yuchuan Dong, Jingjing Chang, Jie He, Hejie Chen, Qiyan Liu, Chunhua Wei, Jianxiang Ma, Yong Zhang, Jianqiang Yang, Xian Zhang

**Affiliations:** ^1^Department of Horticulture, Northwest A&F UniversityYangling, China

**Keywords:** cold stress, high-throughput sequencing, melatonin, microRNA, watermelon

## Abstract

Transcriptional regulation of cold-responsive genes is crucial for exogenous melatonin-mediated cold tolerance in plants. Nonetheless, how melatonin regulates cold-responsive genes is largely unknown. In this study, we found that exogenous melatonin improved cold tolerance in watermelon by regulating expression of microRNAs (miRNAs). We identified a set of miRNAs that were regulated by melatonin under unstressed or cold conditions. Importantly, mRNA-seq analysis revealed that melatonin-induced downregulation of some miRNAs, such as *miR159-5p*, *miR858*, *miR8029-3p*, and *novel-m0048-3p* correlated with the upregulation of target genes involved in signal transduction (CDPK, BHLH, WRKY, MYB, and DREB) and protection/detoxification (LEA and MDAR) under cold stress. These results suggest that miRNAs may be involved in melatonin-mediated cold tolerance in watermelon by negatively regulating the expression of target mRNAs.

## Introduction

Plants have to endure various abiotic stresses due to their sessile life-style. In particular, cold stress is one of the destructive environmental stresses that considerably reduce both yield and quality of fruits and vegetables in tropics and subtropics (Rivero et al., 2001, 2002). Transitory as well as constant cold stress causes damage to cell membranes, which disrupts the balance between water uptake and transpiration, leading to dehydration in shoots. Cold stress decreases the photosynthesis rate by affecting stomatal movement (Foyer et al., 2002), and induces accumulation of reactive oxygen species (ROS) by disrupting electron transport system in both mitochondria and chloroplasts (Suzuki and Mittler, 2006). ROS at high concentration can damage membranes through lipid peroxidation, break DNA strand, and inactivate various vital enzymes (Cheng and Song, 2006).

To survive cold stress, plants have evolved a variety of stress response mechanisms that minimize damage, ensure proper cellular homeostasis, and enable plants to function under stressful condition. Molecular sensors distributed in different cellular compartments can sense any decrease in growth temperatures, and thereby generate secondary signals, such as Ca^2+^, ROS, and inositol 1,4,5-trisphosphate (InsP), and activate different transcriptional regulators, such as basic helix-loop-helix (BHLH), INDUCER OF CBF EXPRESSION (ICE) 1, C-repeat-binding factor (CBF), WRKY, and MYB, via the activation of phosphoprotein kinases, such as calcium-dependent protein kinases (CDPKs) and multiple mitogen-activated protein kinases (Xiong et al., 2002; Mahajan and Tuteja, 2005). Eventually, late embryogenesis abundant (LEA) proteins, chaperones, detoxification enzymes, pathogenesis-related proteins, mRNA/protein-binding proteins, proteinase inhibitors, transporters, lipid-transfer proteins, and enzymes required for osmoprotectant biosynthesis are induced to maintain normal physiological processes including photosynthesis (Janská et al., 2010).

Melatonin (*N*-acetyl-5-methoxytryptamine), a low molecular-weight molecule with an indole ring in its structure, is ubiquitous in living organisms (Hardeland et al., 2011; Tan et al., 2012). In vascular plants, melatonin was first identified in 1995 (Dubbels et al., 1995; Hattori et al., 1995) and numerous subsequent studies have established its important roles in plant growth, development, and defense against various abiotic and biotic stresses, such as salinity, drought, cold, excess copper, and pathogens (Zhang et al., 2014; Arnao and Hernández–Ruiz, 2015). Previously, the primary role of melatonin in stress mitigation was considered as a broad spectrum antioxidant that directly scavenges ROS and/or modulates cellular antioxidant system (Wang P. et al., 2012; Zhang and Zhang, 2014; Manchester et al., 2015). Recent studies have revealed that melatonin could also activate defense systems by regulating the expression of stress-responsive genes involved in signal transduction. For example, exogenous melatonin upregulates the expression of cold-responsive genes such as *C_2_H_2_-type zinc finger transcription factor* (*ZAT*) *10*, *ZAT12*, *CBFs*, *cold-responsive gene* (*COR*) *15*, and *calmodulin-binding transcription activator* (*CAMTA*) *1* under cold stress (Bajwa et al., 2014). Furthermore, Shi and Chan (2014) reported that the AtZAT6-activated CBF pathway might be essential for melatonin-mediated response to freezing stress in *Arabidopsis*. However, the mechanism by which melatonin regulates the expression of stress-responsive genes to activate defense networks is unclear.

MicroRNAs (miRNAs) with a length of 19–25 nucleotides (nt), are highly conserved, endogenous, single-stranded non-coding RNA molecules. An increasing number of studies have demonstrated that miRNAs are important regulators in plant development and stress responses. Generally, miRNAs regulate the response to biotic and abiotic stresses by binding to reverse complementary sequences, resulting in the cleavage or translational inhibition of target mRNAs (Khraiwesh et al., 2012; Sunkar et al., 2012). For instance, *miR398* is downregulated to release its suppression of *CSD1* and *CSD2* in plant responses to oxidative stress (Sunkar et al., 2006). The increased sensitivity of *TamiR159*-overexpressing rice lines to heat stress suggests that the downregulation of *TamiR159*, which targets *TaGAMYB1* and *TaGAMYB2* in wheat, is involved in a heat stress-related signaling pathway, and therefore contributes to heat stress tolerance (Wang Y. et al., 2012). Transcriptional control of the expression of cold-responsive genes is well known, but miRNAs have recently been added to the suite of cold-responsive gene regulatory networks. Various miRNAs that target stress-related genes are significantly up- or downregulated during cold stress in a range of plant species (Khraiwesh et al., 2012; Sunkar et al., 2012).

Watermelon (*Citrullus lanatus* L.), one of the most economically important crops in the world, is highly sensitive to low temperatures (Rivero et al., 2002). In this study, by assessing the watermelon plants in terms of leaf phenotype, the maximum quantum yield of PSII (*F*_v_*/F*_m_), and relative electrolyte leakage (REL) under cold stress, we found that exogenous melatonin could enhance watermelon tolerance to cold stress. To understand the molecular mechanisms of melatonin-mediated cold tolerance in watermelon, we carried out a high-throughput miRNA and mRNA sequencing. The results showed that melatonin up- or downregulated a set of miRNAs under normal temperature or cold stress conditions. Importantly, under the cold stress, the downregulation of some miRNAs, such as *miR159-5p*, *miR858*, *miR8029-3p*, and *novel-m0048-3p*, by melatonin may cause the upregulation of putative target genes involved in signal transduction (CDPK, BHLH, WRKY, MYB, and DREB) and protection/detoxification (LEA and MDAR), suggesting that some miRNAs may be involved in melatonin-mediated cold tolerance in watermelon.

## Materials and Methods

### Plant Materials, Growth Conditions, and Treatments

Watermelon (*C. lanatus* L., cv. Y134) seeds were surface sterilized with 5% sodium hypochlorite (NaOCl) solution for 5 min and then rinsed with running water and distilled water. The sterilized seeds were soaked in distilled water for 8 h and sown directly in pots filled with a mixture of peat/vermiculite (3/1, v/v). Plants were grown in growth chambers with the following environmental conditions: a constant relative humidity of 60–70%, a 12-h photoperiod, 25/18°C (day/night), and a photosynthetic photon flux density of 600 μmol m^-2^ s^-1^. The plants were watered daily and fertilized with Hoagland’s nutrition solution at 1-day interval.

Seedlings at the four-leaf stage were sprayed with 150 μM melatonin solution for 3 days, with distilled water used as the control. The melatonin (Sigma-Aldrich, St. Louis, MO, USA) solutions were prepared by dissolving the solute in ethanol followed by dilution with Milli-Q water [ethanol/water (v/v) = 1/10,000]. Each plant was sprayed with 20 mL of solution. Twelve hours after the third spray of melatonin, the plants were exposed to cold stress (i.e., 4°C) for 36 h with a 12-h photoperiod and photosynthetic photon flux density of 600 μmol m^-2^ s^-1^. Leaf samples were harvested at different time-points after imposition of cold stress, such as at 0, 3, 6, 12, 24, and 36 h to analyze *Cla020078* (*CBF1*) and *Cla020702* (*MYB*) transcript levels, at 6 h to sequence miRNAs and mRNAs, and at 36 h to analyze cold tolerance.

### Analysis of Chlorophyll Fluorescence and REL

The maximum photochemical efficiency of PSII (*F*_v_/*F*_m_) was measured with Portable Chlorophyll Fluorometer (PAM2500; Heinz Walz, Effeltrich, Germany), after the whole plants were dark adapted for 30 min. Minimal fluorescence (*F*_o_) was measured during the weak measuring pulses and maximal fluorescence (*F*_m_) was measured by a 0.8-s pulse light at 4,000 μmol m^-2^ s^-1^. *F*_v_/*F*_m_ was determined using the third leaf of watermelon plants counting from the bottom to up. The calculation of *F*_v_/*F*_m_ was done by the following formula as described by van Kooten and Snel (1990). *F*_v_/*F*_m_ = (*F*_m_ - *F*_o_)/*F*_m_.

REL in the leaves was determined as previously described elsewhere (Zhou and Leul, 1998).

### Library Construction and Sequencing of Small RNA

Eight independent small RNA libraries from plant leaves for four treatments (Control, CK; Melatonin, MT; Cold; Melatonin+Cold, MT-C), with two biological replicates for each treatment, were sequenced. The sequences have been deposited into the NCBI Sequence Read Archive database (SRP078211, SRA438995). Library construction and sequencing of small RNA were performed using a service provider Gene Denovo Co. (Guangzhou, China). Total RNA was extracted using miRNeasy Mini Kit (Cat#217004, QIAGEN GmBH, Germany) according to the manufacturer’s protocol. Total RNA integrity was measured on an Agilent 2100 Bioanalyzer system (Agilent) for quality control. A total of 16–35 nt RNA fragments were excised, purified from a PAGE gel, and ligated with 5′ and 3′ adaptors using T4 RNA ligase. Reverse transcription followed by PCR was used to create cDNA constructs based on the small RNA ligated with 3′ and 5′ adapters. Subsequently, the amplified cDNA constructs were purified from agarose gel, in preparation for sequencing analysis using the Illumina Genome Analyzer (Illumina, CA, USA) according to the manufacturer’s instructions.

### Identification and Differential Expression Analysis of Known and Novel miRNAs

The raw sequences were firstly processed by Illumina’s Genome Analyzer Pipeline software to filter out the adapter sequences, low quality as well as low-copy sequences. Then, the extracted small RNA sequences with 15–26 nt in length were subjected to cucurbit species mRNAs, Rfam^1^ and Repbase^2^ to discard mRNA, rRNA, tRNA, snRNA, snoRNA and repeat sequences. Finally, the remaining unique sequences were analyzed by BLAST against miRBase (Release 21)^3^. Solexa sequences with identical or related (one mismatch) sequences from mature miRNAs were identified as known miRNAs (Meyers et al., 2008).

To identify potential novel miRNAs in watermelon, rest of the unmapped small RNA sequences were searched by BLAST against watermelon genome downloaded from cucumber genome database^4^. The mappable sequences were then folded into a secondary structure using UNAfold software^5^. Only the non-coding sequences which could form a perfect stem-loop structure and meet the criteria for miRNAs prediction (Meyers et al., 2008) were then considered to be a potential novel miRNA candidate.

We chose the miRNAs increased or decreased by more than twofold and *P*-value <0.01 in two treatments as the criterion for a melatonin or cold response. Then, the heat map of differentially expressed miRNAs expression profile was drawn with MultiExperiment Viewer version 4.0 and clustering analysis was performed using a hierarchical clustering method (Eisen et al., 1998).

### Expression Analysis of Predicted Target Genes Based on High-Throughput Sequencing

Eight independent mRNA libraries from plant leaves for four treatments (Control, CK; Melatonin, MT; Cold; Melatonin+Cold, MT-C), with two biological replicates for each treatment, were sequenced. The sequences have been deposited into the NCBI Sequence Read Archive database (SRP078211, SRA438977). Library construction and sequencing of mRNA were performed using a service provider Gene Denovo Co. (Guangzhou, China). After removal of low quality sequences, the clean reads were mapped to the watermelon reference genome, allowing up to one mismatch. The differentially expressed genes were identified using the R package edgeR (Robinson et al., 2010). The expression level of each unigene was calculated and normalized to generate FPKM (fragments per kilobase of exon per million mapped fragments). The false discovery rate (FDR) was used to determine the threshold of the *P*-value in multiple tests. In this study, the FDR < 0.05 and fold change ≥1.5 were used as significance cut-offs of the gene expression differences.

### Prediction of Putative Target Genes of Melatonin- or Cold-Responsive miRNAs

A plant miRNA target prediction server^6^, was used to predict putative miRNA target genes with default settings based on the library of Watermelon genome database, version 1. It reports all potential sequences complementary to an inquiring miRNA sequence with mismatches no more than a specified value for each mismatch type. The minimal weighed score ≤3.0 was applied in the prediction according to scoring schema of miRU by Zhang (2005). The other default settings as follows: length for complementarity scoring (hspsize), 20 bp; target accessibility-allowed maximum energy to unpair the target site (UPE), 25.0; flanking length around target site for target accessibility analysis, 17 bp in upstream/13 bp in downstream; range of central mismatch leading to translational inhibition, 9–11 nt (Brodersen et al., 2008).

### RNA Extraction and Quantitative Real-time PCR Analysis

Two cold-sensitive genes *Cla020078* (*CBF1*) and *Cla020702* (*MYB*) were chose to perform quantitative real-time PCR (qRT-PCR). Total RNA was extracted using a RNA extraction kit (Tiangen, Beijing, China) according to the supplier’s instructions. DNA contamination was removed using a purifying column. One microgram of total RNA was reverse-transcribed using the ReverTra Ace qPCR RT Kit (Toyobo, Osaka, Japan) following the supplier’s instructions. The gene-specific primers for qRT-PCR were designed based on their cDNA sequences, as follows: *Cla020078* (F, AGCAGAGCCCTAACACAGGT; R, AATGGTCTTGAGTTGGG), *Cla020702* (F, GATCCATTGACGGCACTAAC; R, TCGCTACAACGTCCTTCATC), and watermelon β-actin gene (F, CCATGTATGTTGCCATCCAG; R, GGATAGCATGGGGTAGAGCA) was used as an internal control (Kong et al., 2014). The qRT-PCR assays were performed using an iCycler Iq Multicolor PCR Detection System (Bio-Rad, Hercules, CA, USA). PCRs were performed using the SYBR Premix ExTaq II (2×) Kit (Takara, Tokyo, Japan). The PCR conditions consisted of denaturation at 95°C for 3 min, followed by 40 cycles of denaturation at 95°C for 30 s, annealing at 58°C for 30 s, and extension at 72°C for 30 s. The quantification of mRNA levels was based on the method of Livak and Schmittgen (2001).

### Statistical Analysis

The experiment was a completely randomized design with three replicates. Each replicate contained at least 10 plants. Analysis of variance was used to test for significance, and significant differences (*P* < 0.05) between treatments were determined using Tukey’s test.

## Results

### The Effects of Melatonin on Cold Stress Tolerance and Cold-Responsive Gene Expressions in Watermelon

**FIGURE 1:**
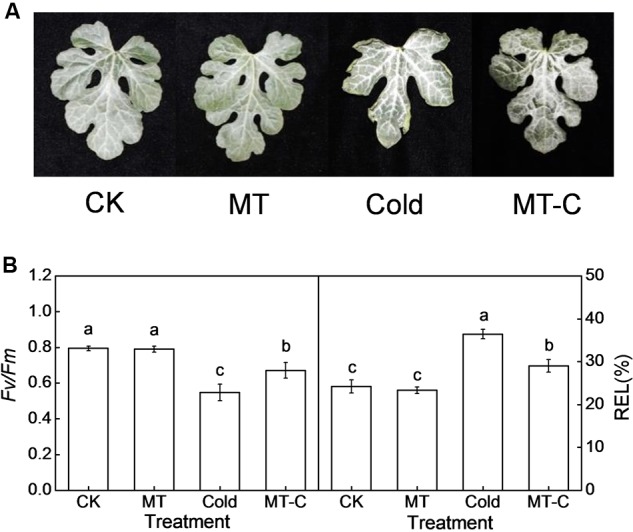
**Changes in **(A)** the leaf phenotypes, **(B)** the maximum photochemical efficiency of PSII (*F*_v_/*F*_m_) and relative electrolyte leakage (REL) as influenced by melatonin or/and cold stress.** Leaves of watermelon (*Citrullus lanatus* L.) seedlings at the four-leaf stage were pretreated with 150 μM melatonin (MT) for 3 days. Then, they were exposed to cold stress (i.e., 4°C) for 36 h. Data of *F*_v_/*F*_m_ are the means of six replicates (±SD). Data of REL are the means of four replicates (±SD). Means denoted by the same letter did not significantly differ at *P* < 0.05 according to Tukey’s test. CK, Control; MT, Melatonin; Cold; MT-C, Melatonin+Cold.

Melatonin plays important regulatory roles in plant defense against various biotic and abiotic stresses (Zhang et al., 2014). In this study, we first analyzed the effects of exogenous melatonin on watermelon tolerance to cold stress. As shown in **Figure 1**, melatonin had a minimal effect on the leaf phenotypes, the maximum quantum yield of PSII (*F*_v_/*F*_m_), and REL at optimal growth temperatures. After 4°C treatment for 36 h, the blade edges of watermelons were wilted, and the *F*_v_/*F*_m_ and REL were significantly reduced and increased, respectively. However, pretreatment with melatonin obviously alleviated cold-induced wilting of blade edges and the reduction and increase in leaf *F*_v_/*F*_m_ and REL, respectively. For example, the *F*_v_/*F*_m_ and REL decreased and increased by 15.45 and 19.82%, respectively in melatonin-pretreated plants after cold treatment, far less than the changes of 31.06 and 50.51% in control plants (**Figure 1**). Taken together, exogenous melatonin significantly enhanced watermelon tolerance to cold stress.

**FIGURE 2:**
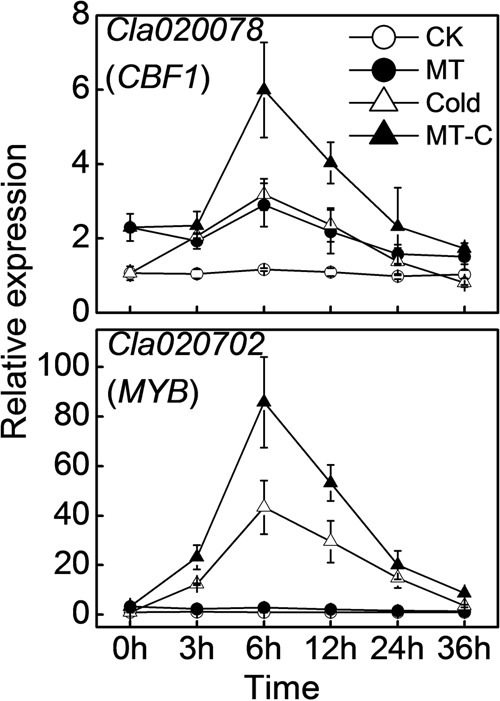
**The dynamic response of the expression of *Cla020078* (*CBF1*) and *Cla020702* (*MYB*) to melatonin or/and cold stress.** The plants were treated as described in **Figure 1** and the leaf samples were harvested at indicated times (h) after cold treatment. Data are the means of four replicates (±SD). Means denoted by the same letter did not significantly differ at *P* < 0.05 according to Tukey’s test.

To analyze the effects of melatonin on the expression of cold-responsive genes and to choose the appropriate time point for miRNA expression profiling, we examined the dynamic responses of *Cla020078* (*CBF1*) and *Cla020702* (*MYB*) to melatonin or/and cold treatment. Transcript levels of *CBF1* and *MYB* were slightly induced by melatonin at optimal growth temperatures (**Figure 2**). After cold treatment (4°C), they were rapidly upregulated, reaching peak levels at 6 h, and subsequently declining to original levels at 36 h. Furthermore, pretreatment with melatonin improved the induction of *CBF1* and *MYB* expression by cold stress. For instance, at 6 h after cold treatment, the expression levels of *CBF1* and *MYB* in melatonin-pretreated plants were upregulated by 5.99- and 85.69-fold, respectively, far more than the increases of 3.18- and 43.38-fold observed in control plants.

### Analysis of MicroRNA Response to Melatonin or/and Cold Based on High-Throughput Sequencing

**FIGURE 3:**
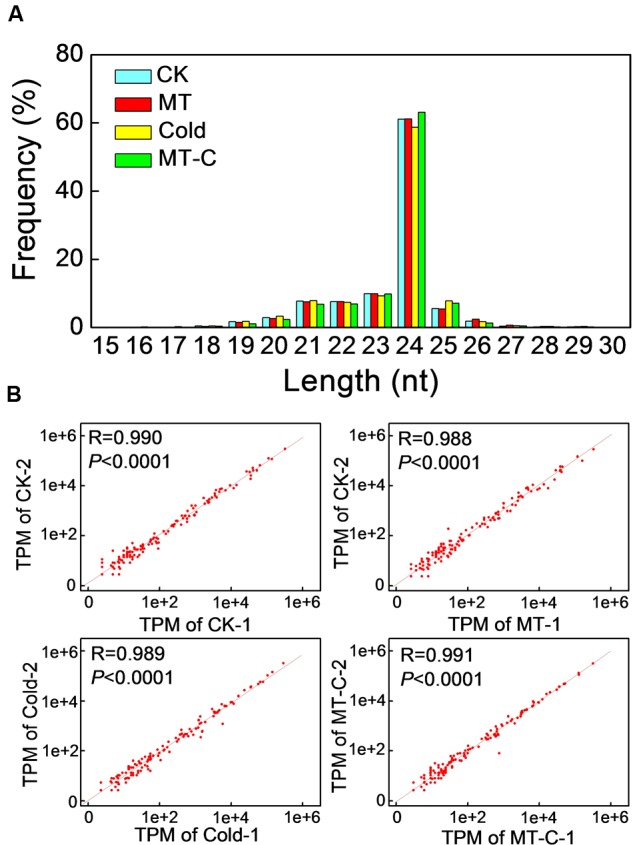
**(A)** Summary of the sequence lengths distribution and **(B)** the correlation between two biological repeats in melatonin or/and cold treatments. The plants were treated as described in **Figure 1** and samples were harvested at 6 h after cold treatment.

To examine whether miRNAs are involved in melatonin-mediated cold response in watermelon, we performed high-throughput sequencing analysis of miRNA in watermelon leaves treated with and without the melatonin and cold stress. A total of 9,704,684/10,831,539 (Control, CK-1/2), 10,124,858/12,383,237 (Melatonin, MT-1/2), 12,812,180/9,718,613 (Cold-1/2), and 9,806,063/11,489,173 (Melatonin+Cold, MT-C-1/2) raw reads were obtained (**Supplementary Table S1**). After the removal of rRNAs, tRNAs, snRNAs, and snoRNAs, a total of 5,327,079/5,914,071, 5,635,109/6,581,846, 6,219,825/5,085,935, and 5,409,138/6,272,887 sRNA sequences for CK-1/2, MT-1/2, Cold-1/2, and MT-C-1/2 were obtained, respectively. The majority of redundant reads were in the range of 19–26 nt, among which the most abundant sequences were 24 nt long in all libraries (**Figure 3A**). To identify miRNAs in watermelon, all sRNA sequences were compared to known plant miRNAs in miRBase Release 21. A total of 440 known unique miRNAs with high sequence similarity to known plant miRNAs were identified (**Supplementary Table S2**). By mapping all unique sRNA sequences to the watermelon genome and predicting the hairpin structures for their flanking sequences, 106 novel miRNAs candidates were identified. The expression level of each miRNA was normalized to generate TPM (tags per million) and the correlation of detected miRNA expression between two biological repeats for each treatment was analyzed. As shown in **Figure 3B**, there was a strong correlation (*R* = 0.99, *P* < 0.0001) between the two biological replicates for all four treatments, suggesting that the sequencing results are highly reliable.

**FIGURE 4:**
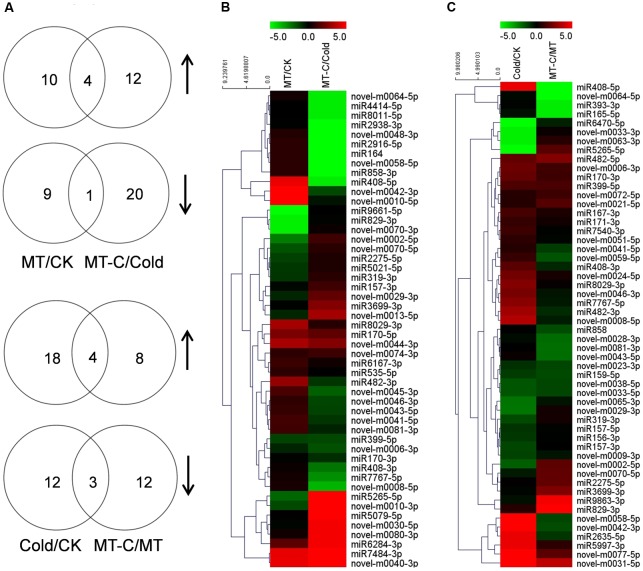
**Analysis of differentially expressed miRNAs induced by melatonin or cold stress.** **(A)** The Venn diagram illustrates number of up- (n-resize) and downregulated (s-resize) miRNAs in comparison of MT/CK, MT-C/Cold, Cold/CK, and MT-C/MT. Up- or downregulation of miRNAs was taken into account when a log_2_ fold change >1, and *P*-value <0.01. Heat map showing **(B)** melatonin or **(C)** cold induced differentially expressed miRNAs and Clustering analysis according to expression pattern. Complete hierarchical clustering carried out using Euclidean distance, color coding was done according to the scale given. Green indicates downregulation and red upregulation. CK, Control; MT, Melatonin; Cold; MT-C, Melatonin+Cold.

A total of 50 miRNAs were significantly up- or downregulated by melatonin under unstressed or cold conditions (**Figure 4**). Among them, four miRNAs such as *miR170-5p*, *novel-m0074-3p*, *novel-m0044-3p*, and *novel-m0040-3p* were upregulated, while one miRNA (*miR399-5p*) were up- and downregulated by melatonin pretreatment, under both unstressed and cold conditions. Ten and nine miRNAs were specifically up- and downregulated by melatonin at optimal growth temperatures, respectively. Twelve and 20 miRNAs were specifically up- and downregulated by melatonin after cold treatment for 6 h, respectively. A total of 54 miRNAs were significantly up- or downregulated by cold stress in control or melatonin-pretreated plants, respectively. Among them, four miRNAs (*miR399-5p*, *miR170-3p*, *miR482-5p*, and *novel-m0031-5p*) and three miRNAs (*miR159-5p*, *novel-m0033-5p*, and *novel-m0038-5p*) were up- and downregulated by cold stress, respectively, in both control and melatonin-pretreated plants. In total, 18 and 12 miRNAs were specifically up- and downregulated by cold in control plants, respectively. While 8 and 12 miRNAs were specifically up- and downregulated by cold in melatonin-pretreated plants, respectively.

### Biological Function Analysis of Differentially Expressed miRNAs via Prediction of Target mRNAs

**FIGURE 5:**
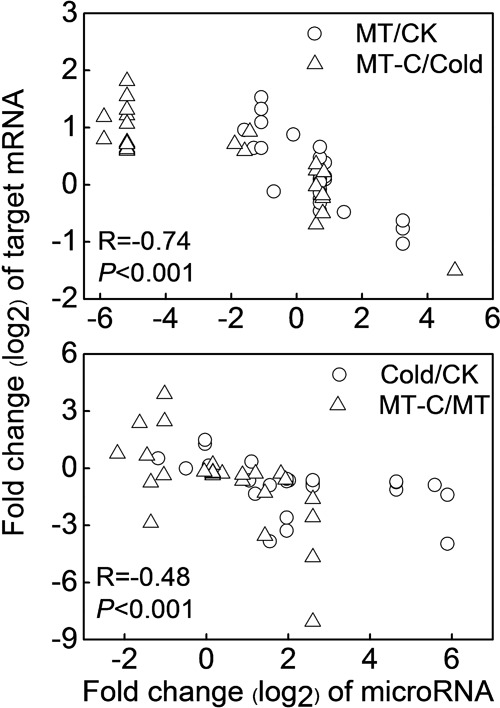
**Correlation analysis between the expression of miRNAs and predicted targets by cleavage in comparison of MT/CK, MT-C/Cold, Cold/CK, and MT-C/MT**.

To examine the biological functions of differentially expressed miRNAs, the miRNA sequences were searched against watermelon genomic sequences using the plant miRNA potential target finder^7^ to predict target mRNAs. miRNAs negatively regulate mRNAs by inducing their cleavage or repressing their translation (Bartel, 2004). In this study, a total of 505 mRNAs were predicted to be cleaved by miRNAs. Then, we performed RNA-seq analysis in watermelon leaves treated with and without the melatonin and cold stress using the high-throughput Illumina Solexa system. Reads from each sample were aligned to the *C. lanatus* reference genome. The expressions of predicted mRNAs in comparison of MT/CK, MT-C/Cold, Cold/CK, and MT-C/MT were analyzed. The expression patterns of only 49 detected mRNAs were opposite with respect to those of 21 corresponding miRNAs (**Table 1**). The negative correlation coefficients between miRNAs and their target mRNAs regulated by melatonin and cold were -0.74 (*P* < 0.001) and -0.48 (*P* < 0.001), respectively, suggesting that these 49 target mRNAs were likely cleaved by the corresponding miRNAs (**Figure 5**). Additionally, 34 miRNAs were searched against a total of 87 potential target mRNAs via translational inhibition (**Supplementary Table S3**). The functions of these mRNAs were annotated in the Cucurbit Genomics Database^8^.

**Table 1:** Potential target mRNAs cleaved by differentially expressed miRNAs.

miRNA	Target	Log_2_ fold change of miRNAs/targets				Description
			
		MT/CK	MT-C/Cold	Cold/CK	MT-C/MT	
miR159-5p	Cla001826	-0.14/0.21	-0.63/0.34	-1.19^∗^/0.53	-1.45^∗^/0.66^∗^	Calcium-dependent protein kinase (CDPK)
miR170-3p	Cla006665	-0.08/-0.04	-1.09^∗^/0.31	2.02^∗^/-0.63^∗^	1.20^∗^/-0.28	GRAS family transcription factor
miR171-3p	Cla006665	0.20/-0.04	-0.31/0.31	1.04^∗^/-0.63^∗^	0.39/-0.28	GRAS family transcription factor
miR399-5p	Cla008388	-1.31^∗^/0.65^∗^	-1.43^∗^/0.93^∗^	1.55^∗^/-3.83^∗^	1.43^∗^/-3.55^∗^	Pentatricopeptide repeat protein 77 (PPRP77)
	Cla005858	/0.23	/-0.18	/-0.88^∗^	/-1.29^∗^	NEFA-interacting nuclear protein NIP30
miR482-5p	Cla014776	-0.05/0.10	0.54/-0.85	2.01^∗^/-0.67	2.60^∗^/-1.61^∗^	Os10g0422600
	Cla013089	/0.43	/-0.97	/-3.27^∗^	/-4.67^∗^	Leucine-rich repeat family protein (LRR)
	Cla009419	/0.12	/-5.36	/2.59^∗^	/-8.06^∗^	Unknown
	Cla016187	/0.86	/-1.31	/-0.56	/-2.57^∗^	C_2_H_2_ zinc-finger transcription factor (C_2_H_2_-ZFTF)
miR858	Cla017745	-0.08/-2.84	-0.11/-0.23	-0.04/1.29	-1.02^∗^/3.90^∗^	Late embryogenesis abundant (LEA) protein like
	Cla017373	/-0.67	/0.30	/1.50^∗^	/2.47^∗^	R2R3 MYB transcription factor
miR2635-5p	Cla021237	7.03/-0.19	-7.96/0.14	4.64^∗^/-0.70^∗^	-1.04/-0.37	Cryptic precocious CRP/Med12
miR2916-5p	Cla001518	0.86/0.39	-5.18^∗^/0.60^∗^	8.51/-1.31^∗^	0.00/-1.09^∗^	DNA primase large subunit
miR5021-5p	Cla010003	-1.08^∗^/0.64^∗^	0.59/-0.02	-0.21/1.13^∗^	1.47/0.46	3-ketoacyl-CoA reductase 2 (KCR2)
	Cla015503	/1.53^∗^	/-0.69	/0.91	/-1.32	RING-H2 zinc finger protein (RING-H2 ZFP)
	Cla001461	/1.33^∗^	/0.25	/0.02	/-1.06	Unknown
	Cla018974	/1.09^∗^	/0.36	/-0.65	/-1.38	J023065D24
miR5997-3p	Cla009517	0.00/-0.12	-0.88/0.33	4.64^∗^/-1.12^∗^	0.88/-0.67	Vacuolar protein sorting-associated protein (VPS) 13
	Cla021487	/-0.09	/0.30	/-0.73^∗^	/-0.35	Cysteine-rich repeat secretory protein (CRRS) 12
miR7540-3p	Cla015655	1.41/-0.15	0.17/1.01	1.19^∗^/-1.33^∗^	-0.05/-0.18	Fasciclin-like arabinogalactan protein (FLA) 2
miR8029-3p	Cla018379	3.29^∗^/-0.17	0.80/0.70	2.60^∗^/-0.68^∗^	0.15/0.18	Monodehydroascorbate reductase (MDAR)
	Cla008020	/0.01	/0.49	/-0.68^∗^	/-0.21	Cyclase/dehydrase
	Cla002580	/-0.10	/0.33	/-0.80^∗^	/-0.36	Lysine-specific demethylase (LSD) 5A
	Cla012681	/-0.29	/0.36	/-0.92^∗^	/-0.27	Pentatricopeptide repeat-containing protein (PPRP)
	Cla004188	/-0.42	/-0.04	/-0.62^∗^	/-0.24	MYB transcription factor
	Cla004079	/-1.03^∗^	/-0.50	/-3.11^∗^	/-2.58	Unknown
	Cla011614	/-0.62^∗^	/-0.18	/-0.27	/0.17	Kinesin like protein
	Cla014313	/-0.77^∗^	/-0.23	/-0.40	/0.13	Peroxidase (POD)
novel-m0006-3p	Cla012226	-0.70/-0.11	-1.59^∗^/0.59^∗^	2.14^∗^/-0.25	1.26/0.46	YABBY
hboxnovel-m0030-5p	Cla014681	-0.11/0.88	4.84^∗^/-1.50^∗^	-8.14/-2.35^∗^	0.13/-4.73^∗^	K^+^ transporter
novel-m0045-3p	Cla003322	1.44/-0.47	-1.89^∗^/0.71^∗^	0.97/-1.11^∗^	-2.40/0.08	Altered inheritance of mitochondria protein (AIMP) 32
novel-m0048-3p	Cla014357	0.71/0.11	-5.18^∗^/0.60^∗^	1.36/-2.46^∗^	-7.90/-1.97^∗^	Glycyl-tRNA synthetase (GARS)
	Cla015456	/-0.46	/0.65^∗^	/-1.87^∗^	/-0.76	Nitrate transporter (NRT)
	Cla022458	/0.48	/1.55^∗^	/-1.03	/0.05	Dehydration-responsive element-binding protein (DREB)
	Cla017676	/-0.24	/1.22^∗^	/-1.47	/-0.01	Kinesin-5 transcription factor
	Cla011198	/0.00	/0.74^∗^	/0.89	/1.63	BHLH transcription factor
	Cla009841	/0.18	/0.73^∗^	/-0.98	/-0.43	Ethylene-regulated nuclear protein ERT2-like
	Cla010176	/-0.08	/0.65^∗^	/0.44	/-0.29	TCP transcription factor
	Cla006034	/0.66	/1.31^∗^	/-0.15	/0.50	Ankyrin repeat-containing protein (Akr1p)
	Cla018866	/-0.14	/0.63^∗^	/-0.49	/0.27	Nucleolar and coiled-body phosphoprotein (NOLC) 1
	Cla016714	/0.10	/1.82^∗^	/-2.37	/-0.66	Os02g0202300
	Cla017355	/0.05	/0.71^∗^	/-1.90^∗^	/-1.23^∗^	WRKY transcription factor
	Cla006039	/-0.31	/1.07^∗^	/-2.55^∗^	/-1.17	Protein forked1
novel-m0058-5p	Cla015802	0.86/0.10	-5.89^∗^/1.19^∗^	5.89^∗^/-3.96^∗^	-1.36/-2.87^∗^	Pentatricopeptide repeat-containing protein (PPRP)
	Cla005115	/0.15	/0.80^∗^	/-1.39^∗^	/-0.74	Chromatin remodeling complex subunit (SWI/SNF)
novel-m0059-5p	Cla019857	1.98/-1.02	-0.82 / 1.01	1.10/0.35	-1.63^∗^/2.38^∗^	TIR-NBS-LRR disease resistance protein
novel-m0070-5p	Cla005664	-1.60^∗^/0.24	0.83/-0.35	-0.50/0.00	1.93^∗^/-0.59^∗^	Nitrate transporter (NRT)
	Cla016054	/0.96^∗^	/0.22	/-0.61	/-1.35	UDP-glucosyltransferase (GTase) 1
novel-m0077-5p	Cla004726	0.00/-0.13	-0.24/0.46	5.58^∗^/-0.87^∗^	1.82/-0.28	IQ-domain 10
novel-m0081-3p	Cla004682	1.25^∗^/-0.46	-0.69/0.17	0.06/0.14	-2.17^∗^/0.77^∗^	Serine/threonine-protein phosphatase (Ser/Thr-PPs)


*^∗^Indicates significant difference.*

**FIGURE 6:**
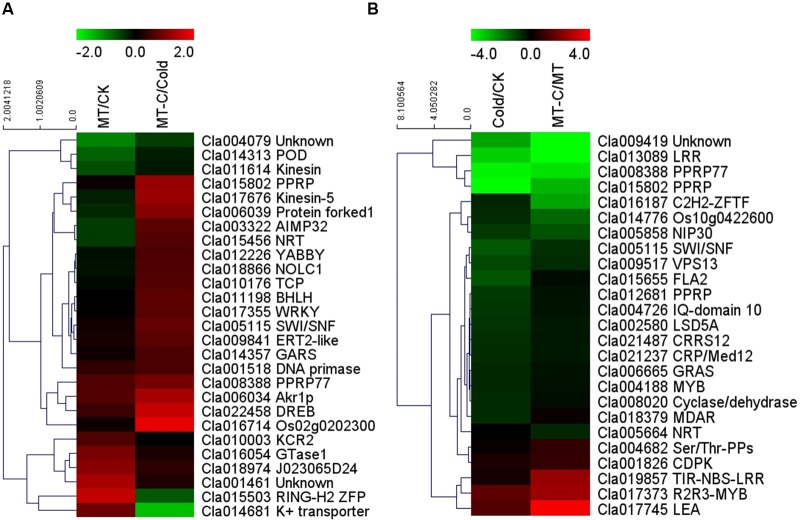
**Heat map showing **(A)** melatonin or **(B)** cold induced differential expression of the potential target mRNAs**.

Some of the 27 potential target mRNAs cleaved by melatonin-responsive miRNAs were induced or repressed by melatonin at optimal growth temperatures, but not under cold stress (**Figure 6A**; **Supplementary Table S4**). Interestingly, most miRNAs including some cold-responsive genes, such as *Cla011198* (*BHLH*), *Cla017355* (*WRKY*), and *Cla022458* (*DREB*, *dehydration-responsive element-binding gene*), were upregulated by melatonin during cold stress, but were not affected by melatonin under unstressed conditions. Most of 25 potential target mRNAs cleaved by cold-responsive miRNAs were repressed or unaffected by cold stress, except for *Cla017745* (*LEA*) and *Cla017373* (*R2R3-MYB*), those were upregulated (**Figure 6B**). However, melatonin pretreatment alleviated cold-induced repression of target mRNAs or improved cold-induced increases in target mRNAs. For instance, *LEA* and *R2R3-MYB* in melatonin-pretreated plants were upregulated by 5.53- and 14.91-fold under cold stress, far more than the changes of 2.82- and 2.44-fold in control plants, respectively. *MYB*, *MDAR* (*monodehydroascorbate reductase*), and *PPRP* (*pentatricopeptide repeat protein*) were significantly repressed by cold stress in control plants, but were minimally affected in melatonin-pretreated plants. Accordingly, a set of potential target mRNAs cleaved by corresponding miRNAs might be involved in the response of melatonin-pretreated plants to cold stress.

**FIGURE 7:**
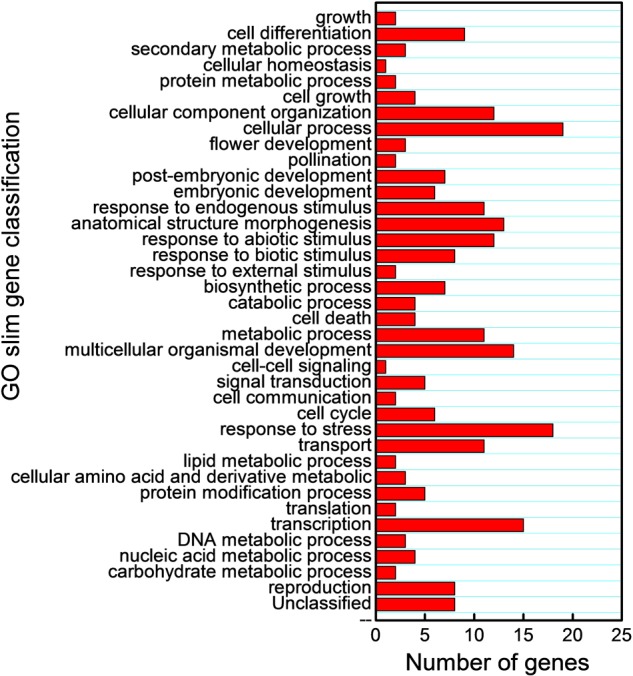
**Gene Ontology classification of potential target genes cleaved by miRNAs based on their involvement in various biological processes**.

We also subjected the predicted target mRNAs to Gene Ontology classification based on their involvement in process in Cucurbit Genomics Database^9^ with watermelon 97103 v1 (**Figure 7**; **Supplementary Figure S1**). The target genes inhibited by miRNAs via cleavage and translational repression were involved in various biological processes, with 19 and 38 mRNAs involved in cellular process regulation, 18 and 31 mRNAs involved in responses to biotic or abiotic stress, and 5 and 10 mRNAs involved in signal transduction, respectively. Thus, a number of potential target mRNAs might be involved in watermelon responses to cold stress by direct or indirect ways.

## Discussion

### Melatonin Induces Cold Stress Tolerance and Cold-Responsive Gene Expressions in Watermelon

In recent years, melatonin has emerged as a focus of research in plant science. It functions in many aspects of plant growth and development, and regulates plant tolerance to abiotic stresses, such as salinity, drought, radiation, excess copper, and chemical stresses (Zhang et al., 2014). Recently, several studies have shown that exogenous melatonin with optimum concentrations enhances cold tolerance in *Arabidopsis* and *Triticum aestivum* L. (Bajwa et al., 2014; Shi and Chan, 2014; Turk et al., 2014), since the initial observation that exogenous melatonin attenuates cold-induced apoptosis in carrot suspension cells (Lei et al., 2004). In the current study, cold stress caused wilted blade edges, decreased maximum photochemical efficiency of PSII (*F*_v_/*F*_m_), and increased REL, which indicates membrane damage (**Figure 1**). Notably, in agreement with previous studies, the adverse effects of cold were significantly alleviated by the application of melatonin. Moreover, melatonin treatment up-regulated two cold-responsive genes *Cla020078* (*CBF1*) and *Cla020702* (*MYB*) in watermelon, which is in line with previous studies in *Arabidopsis* (Bajwa et al., 2014; Shi and Chan, 2014), suggesting that melatonin activates signaling pathways during cold stress via regulation of cold-responsive genes (**Figure 2**).

### Melatonin Affects the Expression of a Number of MicroRNAs under Optimal Growth Conditions or Cold Stress

Transcriptional control of the expression of cold-responsive genes is well known (Chinnusamy et al., 2010), but miRNAs, which could regulate stress responses by controlling the expression of cognate target genes, have recently been added to the suite of cold-responsive gene regulatory networks. Comparative profiles of miRNA expression among various plant species (*Arabidopsis*, *Brachypodium*, and *Populus*) during cold stress revealed similarities and differences in miRNA regulation (Liu et al., 2008; Lu et al., 2008; Zhang et al., 2009). In this study, 440 known miRNAs and 106 novel miRNAs were identified using Solexa high-throughput sequencing (**Supplementary Table S2**). Based on an analysis of differentially expressed miRNAs, we identified a set of miRNAs including 16 known miRNAs and 21 novel miRNAs that were significantly up- or downregulated by cold stress, which exhibited similarities and differences with other species (**Figure 4**; Khraiwesh et al., 2012; Sunkar et al., 2012). The discrepancies in cold-responsive miRNA expression might be attributed to the differential responses of unrelated plant species and variation in experimental conditions and methods. Importantly, pretreatment with melatonin affected the expression of a set of miRNAs under unstressed or cold conditions, and changed the expression of cold-responsive miRNAs. These results suggest that a number of miRNAs are involved in melatonin-mediated enhancement in cold tolerance in watermelon. To the best of our knowledge, this is the first report on the interaction between melatonin and miRNAs in regulation of plant tolerance to stress, although a few studies have reported such interactions in animal cell lines (Lee et al., 2011; Sohn et al., 2015).

### MicroRNAs May Be Involved in Melatonin-Mediated Cold Tolerance by Negatively Regulating Target mRNAs

To examine the functions of melatonin- or cold-responsive miRNAs, we predicted their target mRNAs. We detected 49 mRNAs with opposite expression patterns with respect to their corresponding miRNAs, and inferred that these mRNAs are likely cleaved by the corresponding miRNAs (**Table 1**). Among the 49 potential target mRNAs, several mRNAs were closely associated with plant defense against cold stress by activating signaling transduction or encoding functional proteins. These mRNAs include *Cla001826*, *Cla011198*, *Cla022458*, *Cla004188*/*Cla017373*, *Cla017355*, *Cla017745*, and *Cla018379* which encode CDPK, BHLH transcription factor, DREB protein, MYB transcription factor, WRKY transcription factor, LEA protein, and MDAR, respectively. As Ca^2+^ sensors, CDPKs those can directly bind Ca^2+^, are major players in coupling cold stress signals to specific protein phosphorylation cascades, leading to the activation of downstream cold-responsive genes, such as *ICE1*, a MYC-type BHLH transcription factor (Janská et al., 2010). *ICE1* is activated by low temperatures and triggers *CBF*/*DREB* expression, which promotes the accumulation of cold-responsive gene products such as LEA, antioxidant enzymes, and molecular chaperones (Janská et al., 2010). MYB and WRKY transcription factor gene families have been suggested to play important roles in the regulation of transcriptional reprogramming associated with plant responses to cold stress (Shinozaki et al., 2003; Zhu et al., 2005; Chen et al., 2012). Overexpression of some *MYB* or *WRKY* genes enhances plant tolerance to cold stress and activates the expression of a set of cold-responsive genes such as *LEA*, *DREB2A*, and genes encoding antioxidant enzymes (Dai et al., 2007; Niu et al., 2012; Yang et al., 2012). LEA is important for membrane stabilization and the protection of proteins from denaturation when the cytoplasm becomes dehydrated (Janská et al., 2010). MDAR is an important antioxidant enzyme that is critical to alleviate cold-induced oxidative damage (Chen and Paul, 2002). Importantly, these defense-related genes were significantly induced by melatonin, exhibiting contrasting expression patterns with respect to the corresponding miRNAs, including *miR159-5p*, *miR858*, *miR8029-3p*, and *novel-m0048-3p* (**Figures 5** and **6**). Accordingly, it could be inferred that some cold resistance pathways in melatonin-pretreated plants were promoted by a number of downregulated miRNAs under cold stress.

Some other potential target genes might also be involved in watermelon responses to cold stress by direct or indirect ways. For example, low temperatures can inhibit the uptake and transport of nitrate (Warren, 2009; Leffler et al., 2013; Dechatiwongse et al., 2014). The significantly upregulated expression of *Cla015456* (*NRT*) by melatonin during cold stress suggests that the active transport of nitrate could be helpful in the acclimation of plants under adverse environmental conditions (Zhang et al., 2005). Additionally, the differentially expressed miRNAs were predicted to target a set of mRNAs via translational repression (**Supplementary Table S3**). Some of these mRNAs, such as *MYB*, *MYC*, and *CYP450*, are important in transcriptional regulation and protection in cold responses, respectively. However, whether these predicted target mRNAs are regulated by corresponding miRNAs via translational inhibition requires further studies.

## Conclusion

**FIGURE 8:**
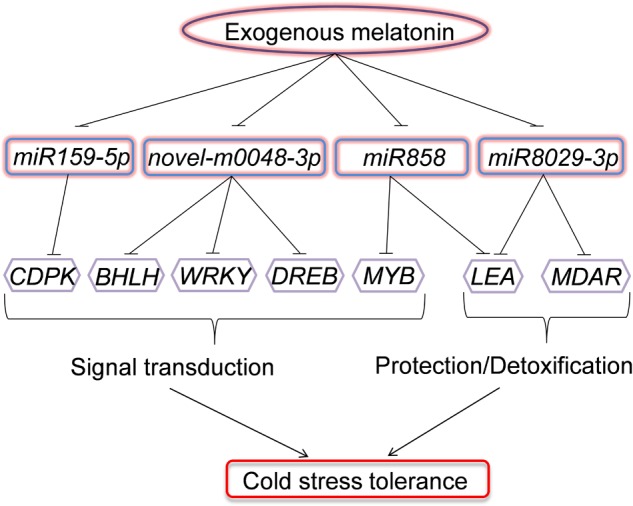
**Model depicting the regulatory pathways of miRNAs in melatonin-mediated cold stress tolerance in watermelon.** Under cold stress, application of exogenous melatonin could downregulate the expression of *miR159-5p*, *miR858*, *miR8029-3p*, and *novel-m0048-3p*. The downregulation of miRNAs results in the upregulation of their target genes involved in signal transduction (CDPK, BHLH, WRKY, MYB, and DREB) or protection/detoxification (LEA and MDAR) and enhance cold stress resistance.

We demonstrated that exogenous melatonin induced watermelon tolerance to cold stress, and this induction was associated with the upregulation of cold-responsive genes. Moreover, we provided evidence for the alteration of the expression profile of a set of miRNAs regulated by melatonin with putative cold-responsive gene targets. In particular, the downregulation of *miR159-5p*, *miR858*, *miR8029-3p*, and *novel-m0048-3p* by melatonin was coincident with the upregulation of putative target genes involved in signal transduction (CDPK, BHLH, WRKY, MYB, and DREB) and protection/detoxification (LEA and MDAR) under low-temperature conditions (**Figure 8**). To our knowledge, these results provide the first evidence for a potential regulatory role of miRNAs in melatonin-mediated cold tolerance in plants.

## Author Contributions

HL and XZ designed research; HL, YD, and JC performed research; HL, JH, HC, QL, CW, JM, YZ, JY, and XZ analyzed data; HL, YD, and XZ wrote and revised the paper.

## Conflict of Interest Statement

The authors declare that the research was conducted in the absence of any commercial or financial relationships that could be construed as a potential conflict of interest.

## Footnotes

1 http://rfam.xfam.org/

2 http://www.girinst.org/

3 http://www.mirbase.org/

4 http://cucumber.genomics.org.cn/

5 http://rna.tbi.univie.ac.at/cgi-bin/RNAfold.cgi

6 http://plantgrn.noble.org/psRNATarget/

7 http://plantgrn.noble.org/psRNATarget/

8 http://www.icugi.org

9 http://www.icugi.org

## Supplementary Material

**FIGURE S1 | Gene Ontology classification of potential target genes of miRNAs with translational repression based on their involvement in process.**

**TABLE S1 | Reads abundance of various classification of small RNAs in control plants (CK) and plants treated by melatonin (MT), Cold, and melatonin+cold (MT-C).**

**TABLE S2 | Identified known and novel miRNAs in watermelon.**

**TABLE S3 | Predicted target mRNAs of differentially expressed miRNAs with translational inhibition.**

**TABLE S4 | The FPKM value of melatonin- or cold-responsive potential target mRNAs in each treatment.**
