# Supplementary figures and images for: High-Throughput MicroRNA and mRNA Sequencing Reveals That MicroRNAs May Be Involved in Melatonin-Mediated Cold Tolerance in Citrullus lanatus L
Source: Front Plant Sci. 2016 Aug 15;7:1231. doi: 10.3389/fpls.2016.01231 (PMC4983558; doi:10.3389/fpls.2016.01231)

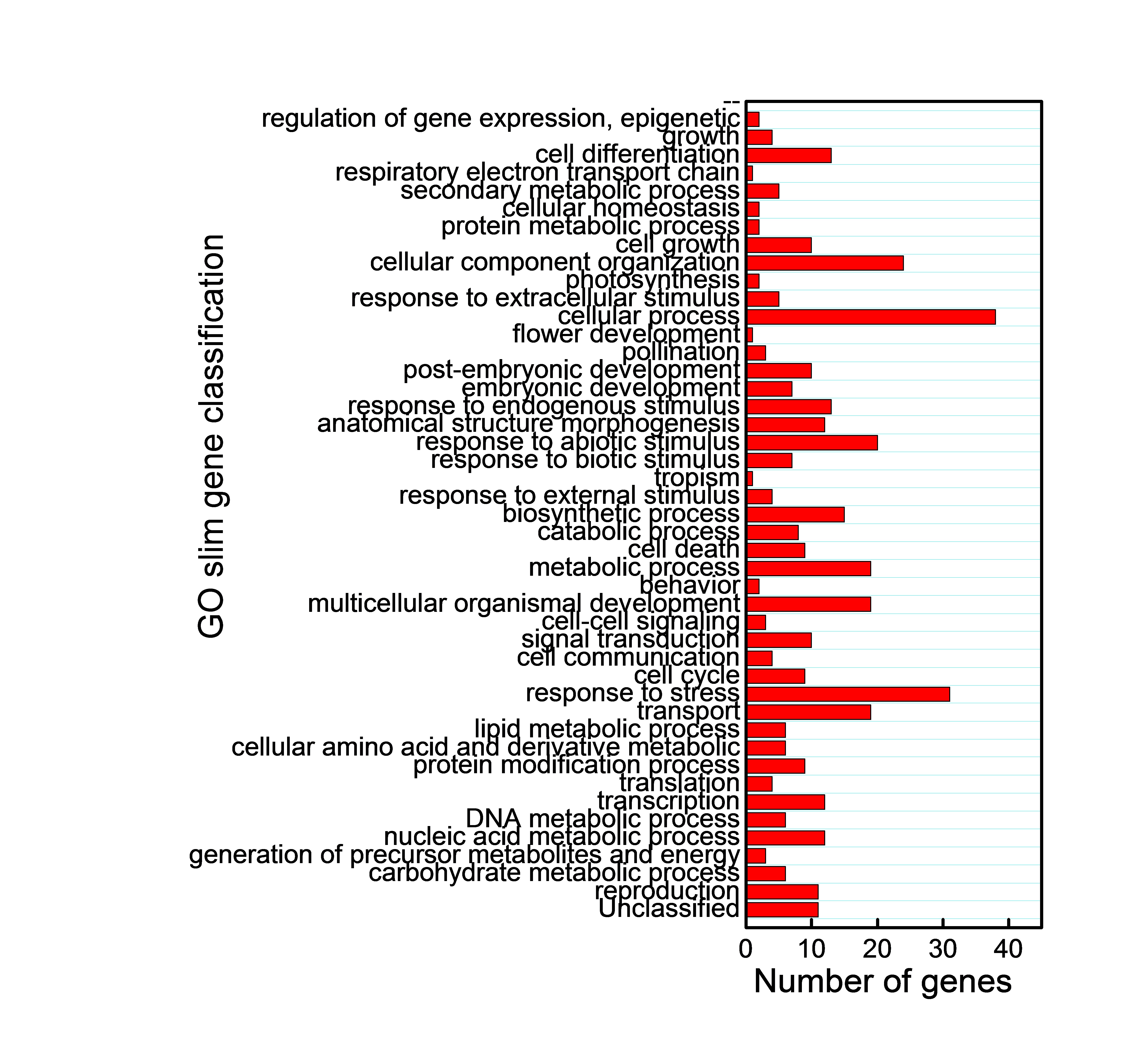

Supplement: Supplementary file 1 [file Image_1.JPEG]
