# Supplementary material for: High-Throughput MicroRNA and mRNA Sequencing Reveals That MicroRNAs May Be Involved in Melatonin-Mediated Cold Tolerance in Citrullus lanatus L
Source: Front Plant Sci. 2016 Aug 15;7:1231. doi: 10.3389/fpls.2016.01231 (PMC4983558; doi:10.3389/fpls.2016.01231)
Supplement: Supplementary file 2 [file Table_1.DOCX]

**Table S1**. Reads abundance of various classification of small RNAs in CK, MT, Cold, and MT-C

| RNA class | CK-1  Raw/Unique | CK-2  Raw/Unique | MT-1  Raw/Unique | MT-2  Raw/Unique | Cold-1  Raw/Unique | Cold-2  Raw/Unique | MT-C-1  Raw/Unique | MT-C-2  Raw/Unique |
| --- | --- | --- | --- | --- | --- | --- | --- | --- |
| Total | 9704684 | 10831539 | 10124858 | 12383237 | 12812180 | 9718613 | 9806063 | 11489173 |
| Clean reads | 9155673/  5367644 | 10205318/  5957942 | 9565945/  5674055 | 11664041/  6631576 | 12104453/  6270188 | 9173989/  5127567 | 9260461/  5448056 | 10870941/  6312139 |
| rRNA | 266390/  28498 | 270496/  30969 | 237976/  27400 | 368698/  34568 | 389007/  33256 | 244733/  28461 | 215378/  27236 | 219731/  26874 |
| snoRNA | 2709/  1326 | 2743/  1392 | 3015/  1430 | 3845/  1604 | 19509/  2202 | 4546/  1391 | 2068/  1186 | 2975/  1419 |
| tRNA | 76006/  8193 | 66551/  9162 | 57064/  7993 | 84633/  11000 | 103646/  11829 | 69884/  9436 | 67782/  8440 | 57886/  8546 |
| snRNA | 4881/2548 | 4514/2348 | 3757/2123 | 5424/2558 | 8087/3076 | 4578/2344 | 3765/2056 | 4579/2413 |
| sRNA | 8805687/  5327079 | 9861014/  5914071 | 9264133/  5635109 | 11201441/  6581846 | 11584204/  6219825 | 8850248/  5085935 | 8971468/  5409138 | 10585770/  6272887 |
