# Supplementary material for: High-Throughput MicroRNA and mRNA Sequencing Reveals That MicroRNAs May Be Involved in Melatonin-Mediated Cold Tolerance in Citrullus lanatus L
Source: Front Plant Sci. 2016 Aug 15;7:1231. doi: 10.3389/fpls.2016.01231 (PMC4983558; doi:10.3389/fpls.2016.01231)
Supplement: Supplementary file 4 [file Table_3.DOCX]

| miRNA | Target | Description |
| --- | --- | --- |
| miR156-3p  miR157-3p  miR159-5p  miR164  miR165-5p  miR170-3p  miR171-3p  miR393-3p  miR408-3p  miR482-5p  miR829-3p  miR3699-3p  miR4414-5p  miR5021-5p  miR5079-5p  miR5265-5p  miR6284-3p  miR6470-5p  miR8011-5p  miR8029-3p  miR9661-5p  novel-m0002-5p  novel-m0006-3p  novel-m0010-3p  novel-m0026-3p  novel-m0029-3p  novel-m0030-5p  novel-m0031-5p  novel-m0033-5p  novel-m0042-3p  novel-m0044-3p | Cla007995  Cla007995  Cla018752  Cla020439  Cla019167  Cla012929  Cla015854  Cla018504  Cla019865  Cla010836  Cla014537  Cla006153  Cla008885  Cla022998  Cla010021  Cla003324  Cla009670  Cla012129  Cla005623  Cla012636  Cla014759  Cla020090  Cla011591  Cla018348  Cla018018  Cla010019  Cla020492  Cla015714  Cla006274  Cla018202  Cla001617  Cla011015  Cla006914  Cla005249  Cla015156  Cla011614  Cla019889  Cla016474  Cla015055  Cla011433  Cla021093  Cla012045  Cla012045  Cla000835  Cla015167  Cla018348  Cla005585  Cla021233  Cla016677  Cla008672  Cla000049  Cla009119  Cla014015  Cla009119  Cla012357 | Peptide transporter (PepT)  Peptide transporter (PepT)  Pentatricopeptide repeat-containing protein (PPRP)  Tetratricopeptide repeat protein (TPR) 5  Calmodulin binding protein-like protein CaM-BP)  Dynamin  EF-hands-containing protein  mRNA clone RTFL01-31-C02  Hypothetical protein  WD-40 repeat family protein  GRAS family transcription factor  Conserved membrane protein YjcL  Serine/threonine protein kinase (Ser/Thr PKs)  Ribosome biogenesis GTP-binding protein YsxC  Cytochrome P450 (CYP450)  Oligopeptide transporter 9  Magnesium chelatase subunit I  PPO-related FMN-binding  Ethylene-responsive transcription factor (ERT) 4  Receptor-like kinase (RLKs)  Protein FANTASTIC FOUR (FAF) 3  Glutathione S-transferase (GST)  Ternary complex factor MIP1  Aspartyl protease-like protein (APL)  Breast cancer (Brca) 2 susceptibility protein  Cytochrome P450 (CYP450)  Protein trichome birefringence-like (TBL) 36  Protein kinase (PKs)  Structural maintenance of chromosome protein (SMC)  NAD-dependent epimerase/dehydratase (Epi)  Protein kinase (PKs)  Pentatricopeptide repeat-containing protein (PPRP)  Alanyl-tRNA synthetase (ARS)  Beta-galactosidase (β-gal)  Glycerol-3-phosphate acyltransferase (GPAT) 2  Kinesin like protein  Microtubule binding protein  Proline dehydrogenase (PDH)  ATPase AAA family protein  Tir-nbs resistance protein  CCR4-NOT transcription complex subunit 1  Acid phosphatase-like protein (APsL)  Acid phosphatase-like protein (APsL)  Pentatricopeptide repeat-containing protein (PPRP)  ADP ribosylation factor  Aspartyl protease-like protein (APL)  Pentatricopeptide repeat-containing protein (PPRP)  Receptor-like protein kinase (RLKs)  Translocase of chloroplast 90  MORN repeat-containing protein  Serine-threonine kinase SepH  RNA-binding protein (RBPs) 5/10  LOB domain protein (LBD) 38  RNA-binding protein (RBPs) 5/10  α/β hydrolase fold |

**Table S3.** Predicted target mRNAs of differentially expressed miRNAs with translational inhibition.

| miRNA | Target | Description |
| --- | --- | --- |
| novel-m0044-3p  novel-m0045-3p  novel-m0046-3p  novel-m0048-3p  novel-m0051-5p  novel-m0058-5p  novel-m0063-3p  novel-m0064-5p  novel-m0065-3p  novel-m0070-3p  novel-m0077-5p  novel-m0080-3p  novel-m0081-5p | Cla010416  Cla023197  Cla011838  Cla019371  Cla011984  Cla019506  Cla003541  Cla002280  Cla013762  Cla010213  Cla004479  Cla010909  Cla014169  Cla008105  Cla022989  Cla022845  Cla019381  Cla009750  Cla006187  Cla005482  Cla020008  Cla000874  Cla007910  Cla013280  Cla019049  Cla003874  Cla003991  Cla009955  Cla009822  Cla021400  Cla005952  Cla020436  Cla002725  Cla008805  Cla009409  Cla009998  Cla019910 | Phosphate translocator (PhoT)  Zinc finger CCCH domain-containing protein 65  Peptide transporter (PepT)  Laccase-22  Queuine tRNA-ribosyltransferase (QueA)  Os01g0678400 protein  50S ribosomal protein L (RPL) 22-like  Cc-nbs-lrr resistance protein  Synaptotagmin (Syt) A  ARGONAUTE 1  ABC transporter G (ABCG) family member 27  Inner membrane protein oxaA  Formation of crista junctions protein (FCJP) 1  U2 small nuclear ribonucleoprotein B  Conserved haloperoxidase-related proteinXylose  Isomerase xylA  Pentatricopeptide repeat-containing protein (PPRP)  CAS1 domain-containing protein  Pentatricopeptide repeat-containing protein (PPRP)  Ent-kaurene synthase 1  26S proteasome non-ATPase regulatory subunit 9  Chaperone ClpB  Ankyrin repeat domain-containing protein (Akr1p) 13C  MYB transcription factor  AT1G74160 protein  Adenosine tRNA methylthiotransferase MiaB  SEC7-like guanine nucleotide exchange family protein  BSD domain-containing protein  Protein phosphatase (PPs) 1 regulatory subunit 7  At5g48610-like protein  Myb family transcription factor  ATP synthase subunit beta (ATP-synβ)  Vesicle-associated membrane protein (VAMP) 7B  MYC2 transcription factor  RING/FYVE/PHD zinc finger-containing protein  Receptor-like kinase (RLKs)  DNA polymerase |
